# A Virtual, 3D Multimodal Approach to Victim and Crime Scene Reconstruction

**DOI:** 10.3390/diagnostics13172764

**Published:** 2023-08-25

**Authors:** Chiara Villa, Niels Lynnerup, Christina Jacobsen

**Affiliations:** Department of Forensic Medicine, University of Copenhagen, Frederik V’s Vej 11, DK-2100 Copenhagen, Denmark; nly@sund.ku.dk (N.L.); christina.jacobsen@sund.ku.dk (C.J.)

**Keywords:** forensic pathology, crime scene investigations, imaging techniques, 3D models, multi-modality approach, virtual reality, 3D printing, research directions

## Abstract

In the last two decades, forensic pathology and crime scene investigations have seen a rapid increase in examination tools due to the implementation of several imaging techniques, e.g., CT and MR scanning, surface scanning and photogrammetry. These tools encompass relatively simple visualization tools to powerful instruments for performing virtual 3D crime scene reconstructions. A multi-modality and multiscale approach to a crime scene, where 3D models of victims and the crime scene are combined, offers several advantages. A permanent documentation of all evidence in a single 3D environment can be used during the investigation phases (e.g., for testing hypotheses) or during the court procedures (e.g., to visualize the scene and the victim in a more intuitive manner). Advanced computational approaches to understand what might have happened during a crime can also be applied by, e.g., performing a virtual animation of the victim in the actual context, which can provide important information about possible dynamics during the event. Here, we present an overview of the different techniques and modalities used in forensic pathology in conjunction with crime scene investigations. Based on our experiences, the advantages and challenges of an image-based multi-modality approach will be discussed, including how their use may introduce new visualization modalities in court, e.g., virtual reality (VR) and 3D printing. Finally, considerations about future directions in research will be mentioned.

## 1. Introduction

In the past two decades, forensic pathology and crime scene investigations have significantly expanded their examination tools with the introduction of various imaging techniques such as CT and MR scanning, surface scanning and photogrammetry. These techniques, as first highlighted by Thali et al. in their 2005 paper [1], have opened new horizons. 

Three-dimensional imaging techniques allow for the non-invasive and non-destructive permanent documentation of individuals and crime scenes. They capture detailed external and internal features of bodies and crime scene evidence, creating high-resolution and precise 3D models. These models can serve as visualization tools or as a foundation for advanced computational analysis.

Adopting an image-based multi-modality and multiscale approach to forensic investigation offers several benefits. Image data can be digitally stored and accessed at any time, facilitating the review of cold cases and enabling virtual crime scene reconstructions. By incorporating 3D models of victims, perpetrators and the crime scene into a single virtual environment, they can be utilized during investigation phases (e.g., hypothesis testing) and court procedures (e.g., intuitive scene and victim visualization) [2,3].

Here, we provide an overview of the techniques and modalities employed in forensic pathology in conjunction with crime scene investigations. We also draw on our own experiences to examine the advantages and challenges of adopting a multi-modality approach. Furthermore, we explore how the utilization of these techniques may lead to the introduction of novel visualization modalities in court, such as virtual reality (VR) and 3D printing. Finally, we touch upon considerations regarding future research directions.

## 2. Three-Dimensional Documentation of the Body: PMCT, PMMR, Surface Scanning and Photogrammetry

Postmortem CT (PMCT) scanning was introduced in forensic institutes worldwide two decades ago [4,5]. Some institutes have dedicated equipment in their autopsy facilities, such as those in Switzerland [5], Denmark [4,6], Australia [7], Japan, [8], the USA, [9,10,11], Norway [12] and China [13,14]. Other institutions rely on equipment available at nearby hospitals, such as those in the UK [15], Italy [16] and Austria [17]. 

PMCT is widely recognized as a complementary tool to traditional autopsy. However, there is still ongoing debate regarding whether PMCT can entirely replace traditional autopsies or if it can be used to enable minimally invasive autopsies [18]. As Rutty and Morgan stated in 2013, “the field is young and the evidence incomplete” [15], and this statement still holds true today. While there have been numerous research efforts to validate PMCT scanning, many of the studies involve single cases or small case series [18,19,20]. More systematic research is needed, including larger cohorts, a comparison with control groups, inter- and intra-observer tests, double-blinded tests conducted by diverse experts and the establishment of standard procedures for result comparison [19,20,21].

The utility of PMCT has been extensively demonstrated (please refer to the reviews in [22,23,24,25]). PMCT scanning is accurate in detecting gas, blood and bone fractures. It enables the rapid identification of atherosclerosis, fat deposition in organs, hemorrhages, some tumors and bowel obstructions as well as indicates lung pathologies. Additionally, PMCT provides access to areas that are not routinely dissected, such as the base of the skull, face, hands and feet. It has proven its worth in disaster victim identification (DVI) [26,27]. 

**Figure 1:**
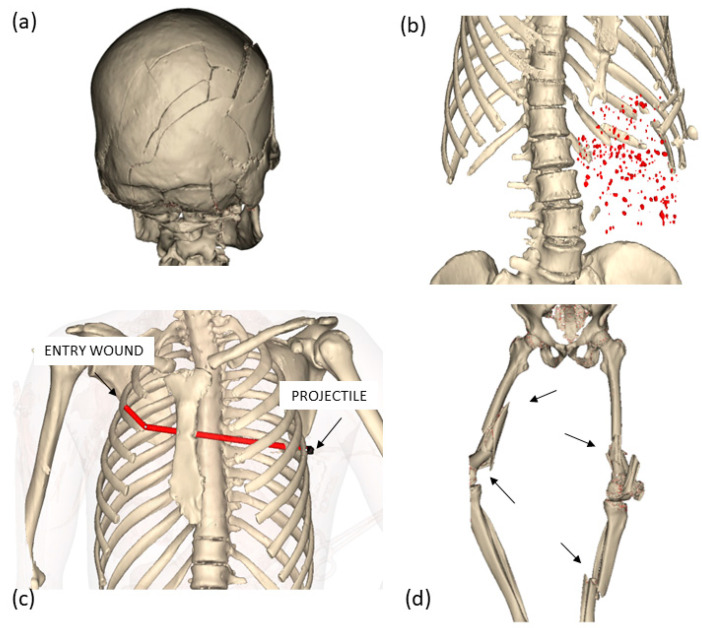
Three-dimensional visualizations from PMCT of several cases: (**a**) blunt force trauma to the head; (**b**) gunshot trauma to the abdomen caused by a shotgun—the pellets are visualized in red; (**c**) gunshot trauma to the thorax—the entry wound, the projectile (as indicated by the arrows) and the bullet path are visualized; (**d**) traffic incident with blunt force trauma to the legs—fractures are indicated with arrows. The 3D models were generated using Mimics software, version 24 [34].

A significant advantage of CT scanning is the ability to generate 3D models of most anatomical and pathological structures, including foreign objects, dependent on CT resolution. The 3D visualizations derived from CT scanning serves as a valuable complementary tool for visualizing fractures and metal objects like projectiles, metal fragments and gunshot pellets (Figure 1). It also allows for 3D visualizations of bullet paths [28,29,30,31]. Precise measurements can be taken using these 3D models, such as in the case of a stab wound or the measurement of fracture height in the bones of the lower extremities (information used for estimating the height of the vehicle that hit the person). Furthermore, the volumes of organs can be accurately assessed [32]. Importantly, these 3D models serve as permanent documentation of the body and can be utilized to present medical evidence in a more intuitive manner [2,3,33].

Postmortem Magnetic Resonance (PMMR) is another imaging modality that was introduced in forensic and clinical medicine in the early 2000s [35,36,37]. This technique is particularly valuable for diagnosing soft tissue lesions, detecting pathology and identifying the presence of air and fluid. However, its use is limited due to cost and time constraints, limited accessibility, technical complexity and challenges associated with postmortem changes which influences image quality [35,36,38,39,40,41]. Similar to PMCT, 3D models can be created from PMMR scans, and, e.g., organ volume [42] and bone thickness [43] can be calculated.

In addition to internal imaging techniques, the accurate and precise documentation of skin injuries is an essential aspect of a comprehensive body documentation. This can be achieved using imaging techniques such as 3D surface scanning and photogrammetry.

Photogrammetry is a technique that allows for the creation of precise and measurable 3D colored models of objects of interest from 2D photographs. It is a versatile tool capable of generating 3D objects, ranging from large crime scenes to fingerprints [44]. The resolution of a 3D model, i.e., its level of details, depends on factors such as the number of photographs, camera resolution and proximity to the object of interest. Photogrammetry applications employ triangulation principles to derive 3D models from 2D photographs (for comprehensive insights, refer to [45]).

Surface scanning is another technique for 3D documentation. Two categories of surface scanners exist: laser scanners and structural light scanners. The fundamental concept once again revolves around triangulation. In the case of laser scanning, a laser line (typically red) is projected onto the object, with the camera measuring its reflection. Since the camera and laser are at a known distance, the scanning software can map the surface of the object, record a 3D scan and create a 3D object. Structured light scanners project pattern of light onto the target object for scanning, replacing the laser line. The camera or cameras then assess deviations in the pattern on the object’s surface and create a 3D model of the object. There are many different surface scanners that vary in scanning range, precision and price.

**Figure 2:**
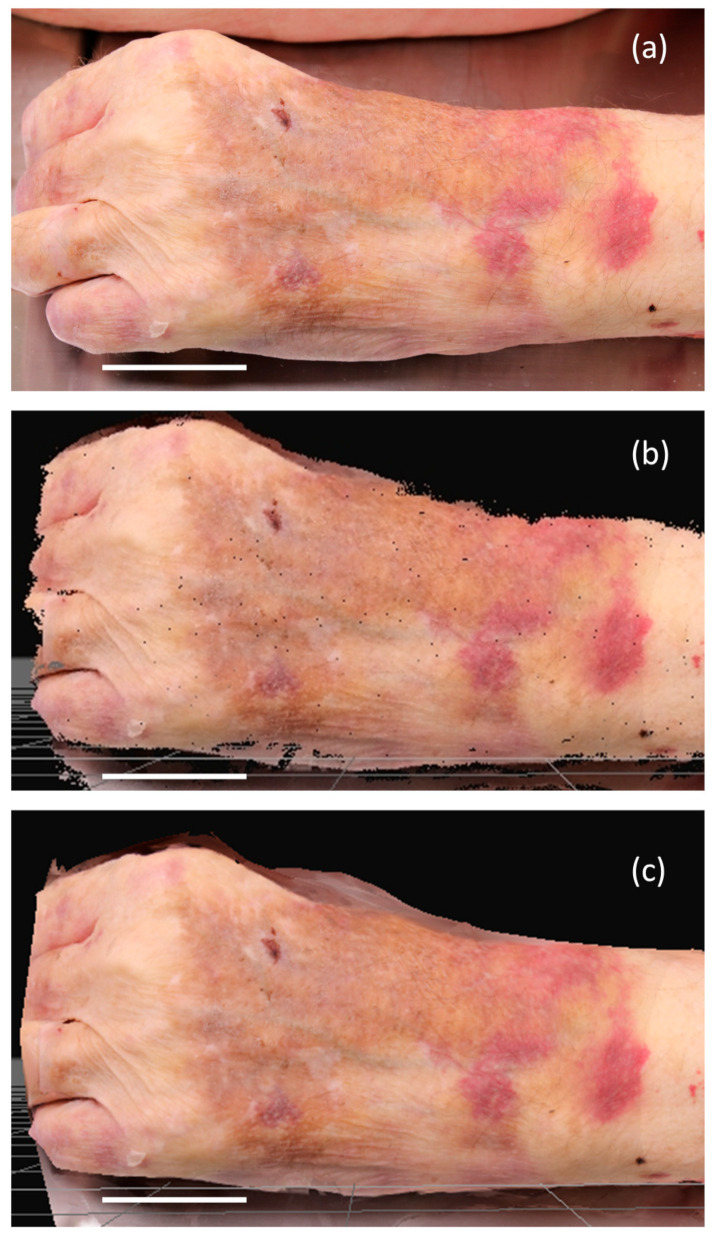
Bruises on a left hand: (**a**) photograph; (**b**) point cloud; (**c**) 3D mesh. The scale bar (white line in the figures) is 5 cm. A canon 5Ds-r with a 50 mm primer was used for the photographs and the software 3DF Zephyr, version 6.507 [59] for creating the 3D models.

These techniques capture detailed information about the skin, including color (texture), with high precision and accuracy. Surface imaging techniques effectively document abrasions, bruises, imprint marks, teeth marks, lacerations and superficial cuts, which may be challenging to detect on PMCT and PMMR scans (Figure 2). Both surface scanners and photogrammetry produce comparable results, each with its own advantages and disadvantages [31,46,47,48,49]. The choice of technique depends on specific needs, budget, area size and desired level of accuracy. In recent years, photogrammetry has received more attention in research. Tests have been conducted using a single camera [47,50,51,52], multi-camera systems [53,54,55,56,57] or videos [58]. These techniques have been applied to deceased [47,51,52,55] and living individuals [54], documenting single lesions, organs or entire bodies.

Recently, LiDAR sensors have been used as a new tool for body documentation [60]. LiDAR, i.e., Light Detection and Ranging, uses an infrared light-pulsed laser to measure distances between objects. LiDAR information is often combined with photographic information to capture texture. LiDAR sensors are now integrated into smartphones (e.g., Apple 12, 13, or 14 or Samsung 23) or tablets (e.g., iPad 11 or 12, or Samsung Galaxy Tab S8). The technique offers greater flexibility and ease of use compared to photogrammetry, as it is less reliant on the operator’s photography skills and provides immediate results, allowing for the evaluation of 3D models directly at the autopsy room. An app called Recon-3D [61], specifically designed for Apple devices, has been successfully tested in forensic contexts [62]. 

### Combining Imaging Modalities

The acquisition of 3D models of both internal and external body structures involves various modalities, software applications and output formats (e.g., mesh surface and point clouds). Combining these diverse 3D data for interactive visualizations and multiscale imaging navigation is a complex task that requires multimodal registration.

The VIRTOPSY group employs a unique approach that involves simultaneously acquiring images of the “naked and cleaned” body using multiple modalities [1,53,63,64]. While this method significantly facilitates the combination of diverse 3D models, its widespread adoption in forensic institutes is yet unlikely. The approach requires performing photogrammetry or surface scanning just prior to or after CT/MR scanning, which may not be feasible for all institutes that rely on hospital equipment. Additionally, it presupposes that the body has already been inspected, without clothing; cleaned; and, if necessary, shaved. However, these steps often occur after the CT scanning. Apart from the local infrastructure and available machinery, procedures leading up to CT/MRI are often dependent on legislation and standards regarding handling of bodies, which can put constraints on which methods and machinery can be used. 

**Figure 3:**
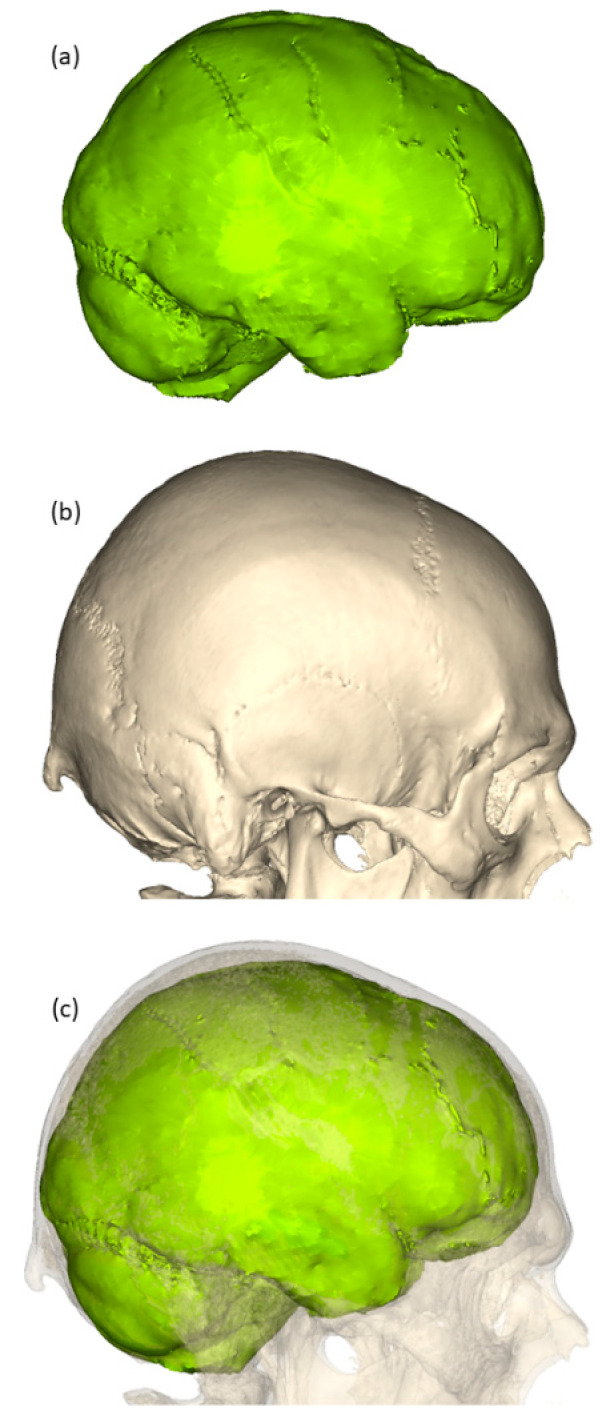
Three-dimensional models of the skull and of the brain from different imaging modalities: (**a**) 3D models from PMMR; (**b**) 3D model from PMCT; (**c**) combined 3D models. The 3D models were generated and aligned using Mimics software, version 24 [34].

Villa et al. [65] proposed a step-by-step procedure for combining 3D data acquired at different times, without reference points on or around the body. An example of combined 3D data from CT and MR scanning can be seen in Figure 3. They utilized the powerful open-source software CloudCompare version 2.8 [66], which is widely used in various applications ranging from industry to forensics and archaeology. 

Another solution, particularly applicable when data are not acquired simultaneously, was proposed by Abreu de Souza and colleagues [67]. They used MeshLab software to align 3D data from DICOM and photogrammetry. Then, they employed MeVisLab software [68] to visualize the inner and outer models along with the DICOM images simultaneously. In recent developments, automatic registration procedures utilizing deep networks have been proposed for aligning 3D point clouds and micro-CT 3D volumes of the same object [69].

## 3. Virtual Animation of the Body: Simulation of the Antemortem Postures

The 3D models generated using different imaging modalities are victim-specific models that accurately represent the victim’s proportions and the exact location of injuries. These 3D models can be animated using animation techniques to reconstruct probable postures at the time of the accident. Various types of computer-generated animation have been employed for forensic reconstruction, including humanoid models [2,70,71,72], biped models [1,73,74,75] and 3D human anatomy models [3]. Each approach has its own advantages and disadvantages.

Humanoid models are quick and easy to create, but they only approximate the victim’s proportions. Software programs like Character Generator [76], Poser [77] and Make Human [78] allow customization of the body proportions of humanoid models, resulting in fully rigged and animated 3D characters. If one wishes to use the actual 3D models of the victim, a biped model can be used. This skeletal model can be easily fitted into the victim’s actual skeleton, but it has a simplified structure with few “joints” and does not account for complex movements. Blender [79], Autodesk 3Dmax [80] or Autodesk Maya [81] can be used for the animation. To achieve accurate simulation of human movement in forensic animation, a large number of joints are required [82]. However, the joint movements are not constrained, and the animation process is time-consuming. Future developments of the human anatomy 3D model should focus on adding inverse kinematic controls and constraints to the joints. This would simplify the movement process, improve accuracy and enable the creation of motion sequences. Moreover, specific 3D models based on sex, age and physical activity level should be developed, as the range of joint motions can vary greatly among individuals [83,84].

Given the extensive use of computer-generated animation in gaming and movies, collaboration between forensic experts and animation/computer science professionals would be beneficial for forensic reconstruction.

## 4. Information of the Perpetrator: Heigh Estimation and Gait Analysis

The perpetrator is a crucial figure in a crime, and it is essential to include their information as well. With an increased number of installed cameras, crimes are more frequently captured on video. In forensic contexts, closed-circuit television (CCTV) camera footage or photographs can provide valuable information about a person’s gait and stature. Gait analysis and stature estimation are important factors in personal identification and are used in many countries as a parameter in forensic anthropological analysis [85,86,87,88,89,90,91,92,93].

The height of a person can be estimated from a photograph or video frame based on stereology principles. If a 3D model of the crime scene is available, obtained through photogrammetry or surface scanning, the images can be oriented, calibrated and scaled using photogrammetry techniques [94,95,96]. By identifying and cross-matching control points in both the photos or 3D models, measurements from the photos or video frames can be obtained. Biped models have also been used to estimate the height of a suspect [91]. Similarly, other anthropological features such as body shape, eye height, shoulder height and foot length can be estimated [97].

The stature of a perpetrator can be significant in understanding the dynamics of an event. For example, was the perpetrator tall enough to cause the stab wound found on the back of the head? Should the perpetrator have been in a higher position to explain the bullet trajectory? These questions can be answered by considering the perpetrator’s stature. Furthermore, such information is important during 3D reconstructions of the chain of the event (see Section 6).

Gait analysis involves assessing how a person’s body moves, typically through walking or running [98,99]. The purpose of a gait analysis is to identify any abnormalities in locomotion and potentially recognize an individual. In the context of a crime, gait analysis can provide information about shoeprints left at the scene and the suspect’s walking and running patterns.

## 5. Three-Dimensional Documentation of the Crime Scene

**Figure 4:**
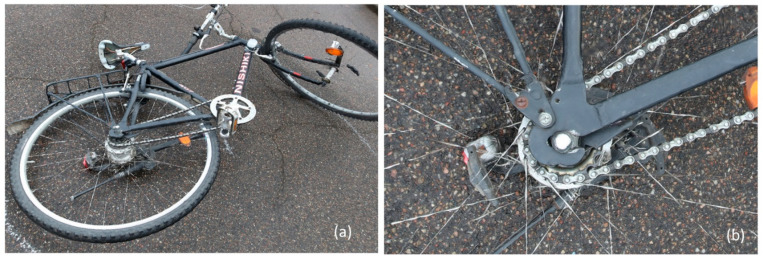
A 3D model (mesh surface) of a bicycle obtained with photogrammetry: (**a**) overview of the bicycle; (**b**) a detailed view of the same bicycle. A canon 5Ds-r with a 24 primer was used for the photographs and the software 3DF Zephyr, version 6.507 [59] was used for creating the 3D model.

Accurate documentation of the crime scene is crucial for police investigations. Surface scanners and photogrammetry have been demonstrated to be highly valuable tools for creating a comprehensive 3D documentation of the entire scene, as well as any relevant evidence such as large or small objects, streets, buildings or victims (e.g., [44,73,74,100,101]). These 3D technologies offer high levels of accuracy and enable the creation of a permanent record of the crime scene (Figure 4). 

Drones, also referred to as unmanned aerial vehicles (UAVs), have been implemented in numerous police services globally and their benefits in the field of forensic investigations have been demonstrated [101,102,103,104].

Research has focused on ensuring the verifiable accuracy of the data and optimizing procedures.

Police authorities from different EU countries (e.g., Germany, Sweden, Italy and Greece) have established networks and regular conferences to share experiences and to stay updated on the latest developments. The EU-funded RISEN project [105] serves as a notable example. RISEN is a consortium composed of research institutions, law enforcement agencies (LEAs) and private companies. Their aim is to develop real-time contactless sensors for optimizing on-site trace detection, visualization, identification and interpretation. The recreated 3D model of a scene incorporates augmented reality techniques to integrate sensor data, collected evidence and identified points of interest. This provides investigators with a realistic and immersive visual environment, facilitating highly detailed investigations.

Annual exercises organized by RISEN allow different LEAs and other partners to test their systems on mock crime scenes, including scenarios such as homicides, clandestine laboratories and terrorist attacks. This approach enables cross-comparison with similar applications and the identification of intersections. The concept was initiated in 1990 by the Police Training Centre in Neuss, Germany, with their “International Police Conference Photogrammetry/Laser Scanning”. The 15th edition of this event took place last year [106]. Specialists in crime scene, along with experts from forensics, science and forensic medicine, attended the conference. The presentations and lectures covered interesting cases and innovations in software and equipment. Importantly, a practical day was included, featuring a different mock crime scene, such as a high-speed traffic incident, train incident or explosion of a tanker vessel.

## 6. Three-Dimensional Reconstructions of the Chain of the Event: Opportunities and Challenges

The events or possible scenarios are often simulated using dummies, rendered by artist [2,107] or reacted with people who resemble the victims [108]. In Denmark, in certain cases, the suspect is asked to reenact the events at the “cleaned” crime scene or a similar setting, together with other persons, e.g., police officers with a similar body stature enacting the victim(s). This process is known as “reconstruction” by the police and is filmed and recorded for use in the judicial process. The defense attorney, prosecutor, police officers and occasionally forensic pathologists observe the reenactment from an adjacent room. During the reconstruction, the suspect shows what occurred during or after the crime, and all observers have the opportunity to ask questions or to request a repeat of the actions. The questioning can lead to changes in the enactment, as the suspect has the freedom to act and respond as they choose, based on their interpretation of how the incidence occurred. This approach encourages dynamic interaction between the participants and may uncover new insights or details about the events under investigation. However, it should be noted that this approach is dependent on the willingness of the parties to actually reenact the scenario and may be biased by the suspect. 

By combining 3D models of the victims, perpetrators and the crime scene, a single virtual 3D environment can be created and visualized. This allows for an accurate visualization of the crime scene, where the spatial relationships between different elements are objectively and faithfully reproduced. Such 3D models can be utilized during both investigation phases and court procedures. This multi-modality virtual approach could be useful for explaining the dynamics of events. Some studies have showed its utility in cases involving gunshot injuries [3,74], traffic incidents [1,73,74] and explosions [100]. It offers a clear and intuitive method to communicate the facts of the case, enabling the audience to better comprehend the circumstances surrounding the crime [2,3]. However, it is crucial not to overlook the numerous challenges associated with the 3D reconstruction of hypothetical scenarios. While presenting an objective visualization of the crime scene is one aspect (3D visualization), delving into the exploration and testing of different scenarios is another (3D reconstruction). Three-dimensional reconstruction of hypothetical scenarios, where the dynamics of the event are virtually simulated, possesses significant influence and can impact the interpretation of objective evidence. There is a potential for subconscious biases to come into play, which can potentially sway the verdict of a jury [109]. It is essential to be aware of these challenges and to take measures to mitigate any potential biases or undue influence during the interpretation of 3D visualizations and simulations in forensic investigations [110]. The critical situation can ensue when the “objective evaluation” of a crime scene is based upon several findings which each have been interpreted in some way, possibly by different experts. This might hamper the objectivity, and scenarios might be interpreted in favor of one or the other party in a judicial setting. The tested scenarios should be able to depict several “realities” and chain of events as it can be difficult to decide which “reality” is the correct/most plausible one. 

Three-dimensional reconstructions involve the interpretation and analysis of the 3D data to reconstruct or understand the events or scenarios that occurred. In this context, several important questions arise which might be answered differently according to national law and judicial practice: Who should generate hypotheses? How many scenarios should be simulated? Should both the prosecutor and defense attorney be given the opportunity to develop reconstructions? Should the process be conducted collaboratively or separately? Should the police provide hypothetical scenarios, while the different parties (prosecutor, defender, forensic experts and forensic pathologists) evaluate them independently? When merging the various 3D information into a single virtual environment, who should be responsible for this task? It is essential to consider that not all crime scene experts are accustomed to working with 3D technologies, and their reports, such as the determination of shooting distances based on standard procedures, may be misinterpreted, e.g., spatially distorted, when incorporated into a 3D environment. Lastly, the animation process presents challenges as individuals move differently, each with their own unique range of motion. How should this variability be considered during the animation process? These are important considerations that need to be addressed in order to ensure accurate and reliable use of 3D technologies in forensic reconstructions and interpretations [111]. A possible solution could involve virtually “recreating” the scene, where different scenarios are visually simulated based on the statements and instructions of suspects, victims or witnesses. As currently practiced in Denmark, all relevant parties should be present during this 3D reconstruction and allowed to ask questions or request re-simulation of specific scenes. To ensure the integrity of the evidence and the accuracy in depicting body movements, an expert 3D team of game designers or programmers should collaborate with the scientific police and forensic pathologists or anthropologists. This collaboration aims to prevent any manipulation of the 3D models and to maintain the fidelity of the evidence. Each virtually reconstructed scenario should be meticulously recorded for potential use in court proceedings. Ultimately, the decision regarding which scenario is deemed the most plausible should be placed in the judicial system.

By employing this approach of virtual “recreation”, different scenarios can be explored, enhancing the transparency and reliability of the investigative process. The use of technology and scientific expertise can contribute to reaching a more informed and unbiased assessment in criminal investigations and legal proceedings.

## 7. Visualization Modalities in Court Proceedings

In court proceedings, reports, 2D photographs and sketches are typically employed to communicate medical evidence and crime scene information. However, the introduction of 3D imaging modalities for evidence recording necessitates the exploration of alternative visualization methods to prevent the loss of information that occurs when converting 3D models into 2D representations or words. Alternative solutions such as “interactive 3D PDF,” immersive 3D technologies and 3D printing have been introduced in many countries around the world.

“Interactive 3D PDF” is a standard PDF file created with Adobe Acrobat, allowing users to view and interact with high-quality 3D models. This solution is advantageous because PDF is a widely used format, and Acrobat Reader is readily available on most computers. As demonstrated by Kottner et al. [112], this approach enables the communication of 3D information while retaining the benefits of a regular 2D PDF. One notable advantage over video is the interactivity of the 3D model.

Urschler and colleagues [113,114,115] have proposed another solution—an innovative computer-aided forensic toolbox. This toolbox provides tools for analyzing, documenting, annotating and illustrating forensic cases using various digital data, including CT and MRI images, 3D models of the body and crime scene evidence, photographs and reports.

Immersive 3D technologies, such as augmented reality (AR), virtual reality (VR) or mixed reality (MR), have been introduced in several countries [116], including, e.g., China [117], Sweden [118,119], the UK [120] and Australia [109]. The VIRTOPSY group introduced its use over eight years ago [121]. These technologies enable investigators to virtually navigate through the crime scene, experiencing different perspectives. For instance, one can sit in the victim’s car and see what the victim likely saw. This type of visualization aids the police in understanding potential scenarios and adds a new dimension to evaluating testimonies [121,122,123]. 

Furthermore, 3D printing has been implemented in several countries. Human skull 3D prints have been used as demonstrative aids in court since 2009 [124], and 3D prints are now commonly employed in various jurisdictions [125,126,127,128].

## 8. Future Directions

The introduction of imaging tools for recording, visualizing and reconstructing the dynamics of events is generally considered advantageous. However, further research is needed to accurately evaluate their practical utility. Qualitative studies should be conducted, focusing on various key actors such as juries, judges, experts and testimonies. The results of these studies could be surprising. For instance, a recent study conducted by Henningsen and colleagues revealed a preference for visualizations on a screen of a 3D virtual model of a traumatic skull over a physically printed 3D model [124]. On the other hand, a study demonstrated that virtual reality (VR) had a positive impact on the verdict decision, as it enabled participants to process spatial information more effectively, resulting in more consistent decision-making [109]. 

Three-dimensional models may be used for further advanced computational analyses. Three-dimensional models of bones obtained from CT and MRI can be used for performing biomechanical analyses. Finite Element Analysis (FEA) allows for an objective assessment of the likelihood of proposed traumatic events, presenting a potentially valuable tool for forensic pathologists in evaluating the force or circumstances of an incident. Previous applications of FEA, including for gunshot trauma [129], traffic incidents [130] and blunt force head injuries in adults, have been attempted [111,131,132]. Furthermore, FEA has been utilized to analyze traumatic events in children [133]. Nevertheless, further studies and testing are necessary to expand our understanding in this area.

Three-dimensional models have also found applications in crime scene investigations that involve quantitative analysis. For instance, they have been utilized in bloodstain pattern analyses in 3D [134,135,136], crash and collision investigations [101,130,137] as well as bullet trajectory analysis [101,137]. These applications leverage the capabilities of 3D models to provide enhanced insights and quantitative data for a more comprehensive analysis of the crime scene.

In the current era of rapid artificial intelligence (AI) development, new possibilities for its application in forensic medicine are emerging [138]. Researchers have conducted numerous studies utilizing AI technology, including sex estimation [139], facial age estimation [140], forensic odontology [141] and gait analysis [142]. Machine learning approaches can make the segmentation of organs and bones from PM imaging modalities quicker, enabling automated detection of fractures [143]. AI can also offer novel methods for analyzing PMCT data, assisting forensic experts in their decision-making [144,145]. AI presents new opportunities for the field; however, it is crucial to establish bridges between different disciplines to facilitate interdisciplinary collaboration between forensic medical experts, engineers and data scientists. Furthermore, it is important to thoroughly test, validate and investigate the advantages and disadvantages before incorporating new methods as routine tools.

Lastly, it is important to highlight some of the challenges associated with using forensic data for research purposes following the implementation of the General Data Protection Regulation (GDPR) in the European Union (EU) in May 2018. The GDPR aims to protect personal data, which includes any information that directly or indirectly identifies an individual within the EU. These rules apply to both living and deceased individuals.

Appropriate security measures must be implemented to safeguard personal data from unauthorized access, loss or disclosure. Encryption, access controls and secure storage are examples of measures that should be considered. Thus, the use of cloud software should be limited or avoided to minimize potential risks. Specific regulations and requirements may vary across jurisdictions and countries. Therefore, consulting legal professionals or relevant authorities who can provide tailored advice based on the specific situation is essential.

## 9. Conclusions

Forensic pathology and crime scene investigations have witnessed significant advancements in examination tools over the past two decades, with the introduction of various imaging techniques. These techniques have evolved from visualization tools to powerful instruments for performing, e.g., virtual 3D crime scene reconstructions and biomechanical analysis. In addition, new solutions for presenting 3D models in court, including interactive 3D PDF, immersive 3D technologies and 3D printing, have been introduced in many countries. 

It is paramount to systematically test, validate and investigate the advantages and disadvantages before incorporating a new method as routine tools to ensure its implementation without compromising the judicial system and introducing bias. Interdisciplinary collaboration between forensic medical experts, engineers and data scientists should be promoted and the involvement of key actors such as juries, judges, defense attorneys, prosecutors and experts, should be encouraged to accurately evaluate the effective and judicially correct utility of the new methods in court.

## Data Availability

Not applicable.
